# Correction: Turn-on fluorescent sensors for Cu-rich amyloid β peptide aggregates

**DOI:** 10.1039/d5sd90046h

**Published:** 2025-12-09

**Authors:** Yiran Huang, Liang Sun, Liviu M. Mirica

**Affiliations:** a Department of Chemistry, University of Illinois at Urbana-Champaign 600 S. Mathews Avenue Urbana IL 61801 USA mirica@illinois.edu

## Abstract

Correction for ‘Turn-on fluorescent sensors for Cu-rich amyloid β peptide aggregates’ by Yiran Huang *et al.*, *Sens. Diagn.*, 2022, **1**, 709–713, https://doi.org/10.1039/D2SD00028H.

The authors regret that a statement confirming the use of live subjects in the study was omitted in error from the published article. This statement is detailed below.

All experiments involving live subjects were performed in compliance with relevant laws and followed institutional guidelines. All animals were handled in accordance with the Guidelines for Care and Use of Research Animals established by the Division of Comparative Medicine and the Animal Studies Committee of Washington University School of Medicine.

The Royal Society of Chemistry apologises for these errors and any consequent inconvenience to authors and readers.

